# Image-Based Single Cell Profiling: High-Throughput Processing of Mother Machine Experiments

**DOI:** 10.1371/journal.pone.0163453

**Published:** 2016-09-23

**Authors:** Christian Carsten Sachs, Alexander Grünberger, Stefan Helfrich, Christopher Probst, Wolfgang Wiechert, Dietrich Kohlheyer, Katharina Nöh

**Affiliations:** Institute for Bio- and Geosciences, IBG-1: Biotechnology, Forschungszentrum Jülich GmbH, Jülich, Germany; University of California Berkeley, UNITED STATES

## Abstract

**Background:**

Microfluidic lab-on-chip technology combined with live-cell imaging has enabled the observation of single cells in their spatio-temporal context. The mother machine (MM) cultivation system is particularly attractive for the long-term investigation of rod-shaped bacteria since it facilitates continuous cultivation and observation of individual cells over many generations in a highly parallelized manner. To date, the lack of fully automated image analysis software limits the practical applicability of the MM as a phenotypic screening tool.

**Results:**

We present an image analysis pipeline for the automated processing of MM time lapse image stacks. The pipeline supports all analysis steps, i.e., image registration, orientation correction, channel/cell detection, cell tracking, and result visualization. Tailored algorithms account for the specialized MM layout to enable a robust automated analysis. Image data generated in a two-day growth study (≈ 90 GB) is analyzed in ≈ 30 min with negligible differences in growth rate between automated and manual evaluation quality. The proposed methods are implemented in the software *molyso* (MOther machine AnaLYsis SOftware) that provides a new profiling tool to analyze unbiasedly hitherto inaccessible large-scale MM image stacks.

**Conclusion:**

Presented is the software *molyso*, a ready-to-use open source software (BSD-licensed) for the unsupervised analysis of MM time-lapse image stacks. *molyso* source code and user manual are available at https://github.com/modsim/molyso.

## Background

The cultivation of microbes in microfluidic lab-on-chip (MLOC) systems is an emerging technology that holds promise to accelerate microbial screening processes enormously. Due to continuous flow and efficient diffusion-based mass transport, all key environmental parameters are precisely controllable, enabling homogeneous cultivation conditions of hundreds or even thousands of cultivation sites operated in parallel [1]. Coupled with live-cell imaging, mono-layer MLOCs offer unique spatio-temporal insights with single cell resolution, which are unobtainable from any conventional bulk technique or flow cytometry [2, 3]. For an overview on state of the art cultivation devices, the reader is referred to recent reviews [4–6].

**Fig 1:**
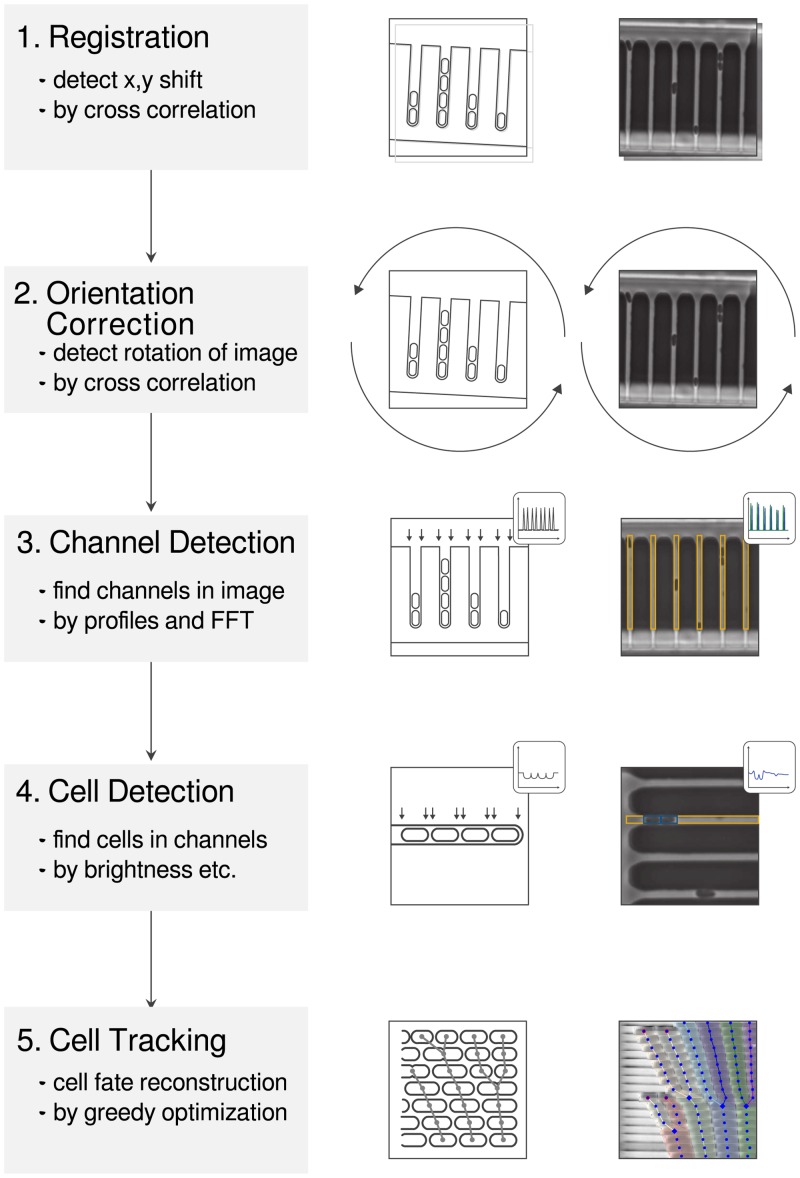
Schematic explanations (central column) and debug output of *molyso* (data from Case Study A, right column).

**Fig 2:**
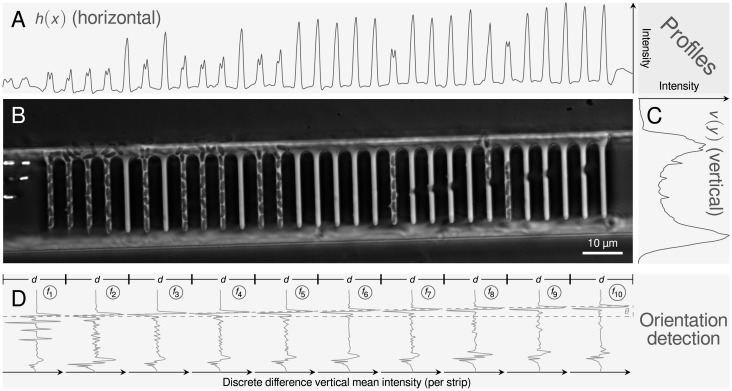
Sample image (region of interest) as acquired from the experimental procedure (**B**). Characteristic mean intensity profiles in horizontal *h*(*x*) and vertical *v*(*y*) direction are shown in **A** and **C**, respectively. Pairwise differences of vertical mean intensity profiles *f* of adjacent strips are used for the orientation correction (**D**). Indicated by the dotted line, main features of the profiles are apparently shifted depending on the rotation of the image. This shift is used to find the orientation correction angle *θ*.

The *mother machine* (MM) was originally designed by Wang et al. [7] for the investigation of *Escherichia coli* cells, but has become increasingly popular for studying various microbes at single cell level. Here, one “mother” cell is trapped per growth chamber, a 1D channel whose width matches the width of a cell. While the mother cell continuously divides at the dead-end of the channel, it pushes its progeny towards the open channel end where it gets flushed away by the nutrient supply stream (cf. Figs 1 and 2B). That way, the mother cell is only in contact with its latest progeny, which minimizes the influence of surrounding cells or medium gradients. This enables long-term experiments with a single cell over many generation times under precisely controlled conditions. Combined with automated high-resolution microscopy and fluorescent labeling, the MM is ideally suited to resolve the phenotypic heterogeneity of bacterial growth [8] and response upon treatment with antibiotics [9] or to address the long-discussed question of bacterial division control (i.e., “timer” vs. “sizer” [10]). Recently, technical adaptions have been described, such as an in-depth fabrication protocol [11], or a chemostat variant with two open ends [12] that further broadens the applicability of the MM.

While the technical setup is comparably easy, the time-lapse data load of long-term experiments can become quite high (≈ 30–50 GB per day). Several automated analysis tools for the evaluation of 2D mono-layer micro-colonies have been developed (e.g., CellProfiler [13], MicrobeTracker [14], Schnitzcells [15] or TLM-Tracker [16]). These tools are, however, not suited for the analysis of MM images, because they are tuned to resolve colony growth patterns. Additionally, the proximity of device structures and cells cannot be well resolved with these tools (see S1 Fig). Recently, two semi-automated solutions tailored for the MM have been described that improve the analysis quality substantially in terms of segmentation and tracking: the ImageJ plugin (*mmJ*) by Arnoldini et al. [9] available at https://github.com/ccg-esb/mmJ and a framework applying joint segmentation and tracking techniques, interactively prompting for user curated corrections [17]. Still, the analysis of millions of division events in thousands of channels per MLOC with such semi-automated approaches can amount to significant manual processing efforts that are infeasible for routine operation.

We present a ready-to-use image analysis pipeline, specifically tailored to the automated processing of time lapse microscopy videos generated in high-throughput MM experiments. Cellular descriptors with single cell resolution (e.g., cell length and growth rate) are accessible, while retaining the full cell history and lineage information as well. This makes quality control of the analysis results a fairly easy task. In contrast to earlier approaches [7], our approach solely relies on phase contrast information for cell segmentation, leaving the fluorescence channels free for implementing biologically meaningful read-outs (e.g., single cell biosensors). The pipeline, implemented in the software *molyso*, is evaluated with two case studies: a small-scale data set for ground truth comparison, as well as a more comprehensive data set with fluorescent biosensor readout. *molyso* is tested with different MM designs and benchmarked with a large-scale dataset (>1 mio cell sightings with ≈ 40,000 division events). Therewith, the automated image analysis with *molyso* speeds up the process of data examination and raises the applicability of the MM as routine phenotypic screening tool.

## Image processing pipeline

The analysis flow is shown in Fig 1. After registration and rotation (steps 1 & 2), the overall structure is detected first, followed by its channels (step 3). Finally, the individual cells within the channels are identified (step 4). In this way, the amount of image data to be passed to the next step is reduced. Steps 2–4 are performed independently for each image of the acquired sequence. This allows for efficient parallelization. Finally, the cell tracking, i.e., finding the relationship between detected cells on successive frames *t* and *t* + 1, is performed to reconstruct the total cell lineage (step 5).

Images are acquired with phase contrast microscopy (cf. S1 Appendix for details), where *I*_*t*_(*x*, *y*) represents the gray level pixel intensity at position (*x*, *y*) at time point *t* = 1, … *T*. The horizontal *h*_*t*_(*x*) and vertical *v*_*t*_(*y*) mean intensity profiles of a *W* × *H* pixel image *I*_*t*_ are defined as follows:

$$\begin{array}{l} h_{t}\left(x\right)=\frac{1}{H}\sum_{y=1}^{H}I_{t}\left(x,y\right),\;x=1,\ldots ,W\\ v_{t}\left(y\right)=\frac{1}{W}\sum_{x=1}^{W}I_{t}\left(x,y\right),\;y=1,\ldots ,H \end{array}$$

Smoothing is applied to profiles in the following processing steps: orientation correction, channel and cell detection. When referred to smoothing, a convolution of the signal with a Hamming window [18] is performed.

### Registration

Stage or microfluidic device movements during image acquisition lead to spatial displacements in (*x*, *y*)-direction that need to be properly corrected before further image processing. To this end, a shift vector ${\vec{\chi }}_{t}$ is calculated by cross-correlating (⋆) 1D mean intensity profiles in horizontal (Fig 2A) and vertical (Fig 2C) direction, rather than using the whole 2D image (Fig 2B). Here, the first image (*I*_1_) of a time lapse series acts as reference for the calculation of the shift vectors ${\vec{\chi }}_{t}$ of all subsequent frames:

$${\vec{\chi }}_{t}=\left(\begin{array}{c} \underset{x}{arg max}\left(h_{1}\left(x\right)⋆h_{t}\left(x\right)\right)\\ \underset{y}{arg max}\left(v_{1}\left(y\right)⋆v_{t}\left(y\right)\right) \end{array}\right)$$

By exerting the shift vectors to the original images, displacement corrected images are derived. For ease of notation, we retain the introduced notation for transformed images. Also, the image index *t* is dropped in the remainder of the text if the operations are applied all time points of the sequence.

### Orientation correction

MM devices are formed by rectangular structures. To facilitate efficient processing, the structure and individual channels have to be rotated, such that regions of interest are rectangular and can be cleanly cropped at pixel borders. For instance, mean intensity profiles have a better signal-to-noise ratio if the structures are orthogonally oriented (cf. Fig 2B). Hence, a properly oriented MM structure lies horizontally within the image, aligned parallel to the image borders, with cells growing in vertically oriented channels. The structure’s rotation angle is then determined as follows: The image is horizontally sliced into *N* equally sized strips of width *d* = *W*/*N*. For each strip (*n* = 1, …, *N*), the difference of its smoothed vertical mean intensity profile is calculated (cf. Fig 2D):

$$f_{n}=v_{n}\left(y\right)-v_{n}\left(y-1\right),\;y=2,\ldots ,H$$

Each adjacent profile pair (*f*_*n*_, *f*_*n*+1_) is then cross-correlated to determine the shift *s*_*n*_ of the *n*-th strip:

$$s_{n}=\underset{y}{arg max}\left(f_{n}\left(y\right)⋆f_{n+1}\left(y\right)\right),\;n=1,\ldots ,N-1$$

Ideally, these shifts *s*_*n*_ are identical for all strips. Artifacts and aberrations in the image, however, may lead to outliers. To remove such outliers, only shift values *s*′ deviating less than two standard deviations (*σ*_*s*_) from the median ($\tilde{s}$) are taken into account for the calculation of the rotation angle (*θ*):

$$\begin{array}{l} s^{\prime }=\{s_{n},n=1,\ldots ,N-1:|\tilde{s}-s_{n}|<2\cdot \sigma_{s}\},\\ \theta =\text{arctan}\frac{\bar{s^{\prime }}}{d} \end{array}$$

Subsequent steps are then performed with the image rotated by the detected angle *θ*. Compared to more elaborate approaches such as Hough transform [19], which is computationally expensive and needs neat edge detected images, mean intensity profiles have proven to be faster and provide more robust results.

### Structure & channel detection

Before detecting the individual channels, the arrays’ vertical extent is determined. Due to its repetitive design, it is straightforward to identify the MM structure in the frequency domain: the horizontal mean intensity profile *h* is used as input signal of a 1D fast Fourier transform to determine the pixel interval *i*_C_ of the channels from the dominant frequency in the spectrum $\hat{h}$ (i.e., the strongest peak in S2A Fig). To find the vertical position of the channel structure, the image is split in vertical direction into strips and a connected region of strips is determined, where the strip’s dominant frequency (cf. S2A Fig) matches the dominant frequency of the complete image. This frequency-based approach is robust against local inhomogeneities in microscopy illumination as it acts as a bandpass filter.

After cropping the image accordingly, the individual channels are located. Due to the optical properties of phase contrast microscopy, channels appear brighter in intensity than the surrounding solid MM device material. The intensity gradient implies that the derivative of the mean horizontal intensity profile (calculated by finite differences) has a zero crossing at mid-channel, a positive peak on the left and negative peak on the right side of each channel (cf. S2D Fig). Thus, the derivative signal *h*′ can be split into “left” *c*_*l*_ (only positive values) and “right” *c*_*r*_ (only absolute negative values) signals (cf. S2E Fig):

$$c_{l}\left(x\right)=\frac{1}{2}\left(h^{\prime }\left(x\right)+\left|-h^{\prime }\left(x\right)\right|\right),\;c_{r}\left(x\right)=\frac{1}{2}\left(\left|h^{\prime }\left(x\right)\right|-h^{\prime }\left(x\right)\right)$$

Cross-correlating the two signals yields the width of each channel *w*_C_:

$$w_{\text{C}}=\underset{x}{arg max}\left(c_{l}\left(x\right)⋆c_{r}\left(x\right)\right)$$

A reference “channel signal” *a* is generated by setting a zero signal to one at the intervals of the channels, once at the left (zero phase shift) and the right edge (phase shift equals *w*_C_) of each channel:

$$a\left(x\right)=\left\{\begin{array}{cc} 1, & \left(x\;\mathrm{mod}\;i_{\text{C}}=0\right)\vee \left(\left(x-w_{\text{C}}\right)\;\mathrm{mod}\;i_{\text{C}}=0\right)\\ 0, & \text{else} \end{array}\right.$$

The reference signal *a* is cross-correlated with the summed “left” and “right” signals *c*_*l*,*r*_, yielding the phase *ϕ*, the shift at which channel structures appear in the image relative to the reference signal:

$$\phi =\underset{x}{arg max}\left(a\left(x\right)⋆(c_{l}\left(x\right)+c_{r}\left(x\right))\right)$$

With the phase *ϕ*, a shifted artificial signal (*a*(*x* − *ϕ*)) is constructed that matches the channels in the image. This signal is used as weighting factor by element-wise multiplication with the sum of the left and right signals, yielding *h*_*τ*_, a robustified channel signal:

$$h_{\tau }\left(x\right)=a\left(x-\phi \right)\cdot \left(c_{l}\left(x\right)+c_{r}\left(x\right)\right)$$

The border (i.e., channel free regions) is found by thresholding *h*_*τ*_, as the introduced weight signal amplifies channels and attenuates non-channel regions. This way, each individual growth channel can be precisely cropped out of the image. The next task is the detection of the single cells within the growth channels.

### Cell detection

Compared to cell detection in free-form environments, the situation is less difficult in tight-fitting MM growth channels: the cells are neatly oriented along one axis without overlapping. Nonetheless, ubiquitous uneven illumination and very low signal-to-noise ratios pose notorious problems for the cell detection. Aiming at a robust and fast solution, instead of contours rectangular bounding boxes are detected with their width matching the channel’s width. As a result, cells per channel are described by 1D coordinate pairs.

**Fig 3:**
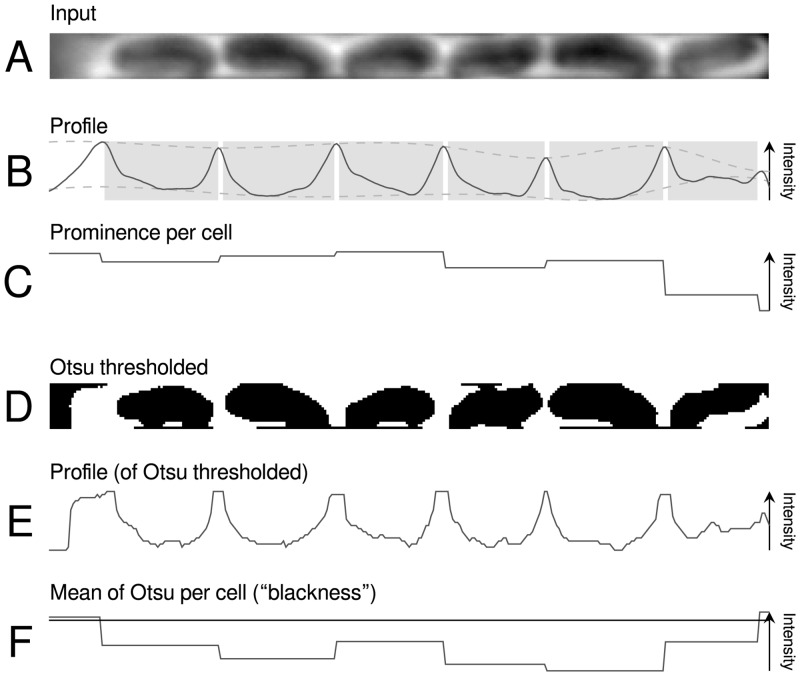
For a crop of a single growth channel (**A**) its Otsu binarization (**D**) is determined. In **B** and **E** the mean intensity profiles of both images are shown. The gray boxes in **B** denote detected cell borders, the splines fitted to the profile maxima and minima used for prominence calculation are indicated by dashed lines. Prominence per cell (**C**) and Otsu mean per cell (**F**) are step functions. The blackness threshold for candidate filtering is denoted by a horizontal line; the threshold for prominence is not included as it is much lower.

Cells are detected in a two step process: First the input channel image (Fig 3A) is used to calculate an intensity profile, which is baseline corrected by subtracting a highly smoothed version of itself (smoothing window length = profile length), and smoothed again, yielding a cleaned intensity profile (Fig 3B). This cleaned intensity profile is used to identify local extrema by a sliding window approach. Pairs of adjacent maxima yield the cell candidates.

Second, these candidates are filtered to exclude false positives. Because methods relying on single techniques were often found to fail in test data sets but correct cell identification is crucial for extraction of meaningful biological information (e.g., cell length or division times), a multi-filter approach was chosen. The criteria to be met are (cf. Fig 3): (i) a cell must have a certain length (e.g., 1 *μ*m); (ii) the “blackness” of a cell must be below a threshold. Here, blackness of a cell is defined as the mean of its extent in the mean intensity profile as calculated by Otsu binarization of the channel image [20]. We found a threshold of 50% to be appropriate; (iii) the “prominence” of a cell must be above a threshold. Prominence is defined as the difference between two splines, one fitted to the maxima and one to the minima of the cleaned profiles (Fig 3B, dashed lines). After this step is performed for every channel image for every time point, the following data has been gathered: a list of channel positions, each with a list of cells, as defined by their upper and lower boundaries.

### Cell tracking

Between adjacent frame pairs, a cell experiences one of these fates: i) the cell remains the *same* (S), possibly it grows or moves (S3A Fig); ii) the cell *divides* (D) into two individual daughter cells (S3B Fig); iii) the cell is *lost* (L), e.g., due to cell lysis or because it is flushed out of the channel, and iv) the cell appears *de novo* (N), e.g., at the initial seeding phase or by entering an empty channel from a neighboring channel. The tracking of cells is formulated as optimization problem as described before [21, 22]. To reconstruct the (likely) lineage from the single cell snapshots, customized costs (*c*) are assigned to all possible cell-cell linkages in consecutive frames where the costs relate to the biological (un)likeliness of the linkages. These costs depend, for instance, on the distance traveled by a cell as compared to its past trajectory or the cell’s length as compared to its former elongation rate. Generally, costs for appearing/disappearing linkages are higher than those for movements and divisions. The individual cost functions are detailed in S1 Table.

The optimal tracking solution is then found by solving a combinatorial cost minimization problem for each frame. Given *J* detected cells in frame *t* and *K* cells in frame *t* + 1, the assignments between the cells are represented by two binary *J* × *K* matrices, the *same* (*S*) and the *division* (*D*) matrix. Furthermore, two 1 × *K* matrices, the *new* (*N*) and the *loss* (*L*) matrix, are defined (as these fates represent no assignment). Correspondingly, cost matrices *C*_{*S*,*D*,*N*,*L*}_ are built applying the cost functions to weight the possible assignments (for details see S1 Table). Finding the best assignment between two frames can then be formulated as constrained linear programming (LP) problem (∘ denotes element-wise matrix multiplication):

$$\begin{array}{ll} \text{min} & \sum_{k}^{K}({\left(c_{N}\cdot N+c_{L}\cdot L\right)}_{1k}+\sum_{j}^{J}{\left(C_{S}\circ S+C_{D}\circ D\right)}_{jk})\\ \text{s}.\text{t}. & \sum_{j}^{J}{\left(S+\frac{1}{2}D\right)}_{jk}+N_{1k}+L_{1k}=1for\;all\;k\in \{1,\ldots ,K\},\\ & (S+D)\;\text{is in reduced row echelon form} \end{array}$$

Herein, the first constraint ensures that cells have exactly one fate, while the second constraint acertains that the cells do not change their order in the channel. The LP problem is solved using a custom iterative greedy approach. Least-cost fates are picked until all cells are assigned. Once a solution has been found for a pair of frames, the reconstructed lineage of the cells is added to the overall lineage tree and the process is repeated until all time points have been tracked.

Finally, to visualize the tracking results, a *kymograph* is generated, aligning the (sub-)images for each channel in ascending frame order. As an overlay, cell fates are shown by lines connecting the cells’ centroids using one color per track, making tracking and division events easily visible.

## Results & discussion

The processing steps are implemented in the software *molyso*. *molyso* is written in Python 3, as such platform-independent and uses only NumPy [23] and SciPy [24] libraries for basic functionality. Input data in ome-tiff format is expected which can be generated from various proprietary formats using the Bio-Formats command line tools [25]. The plotting library matplotlib [26] is used for result visualization. Optionally, OpenCV [27] can be used to improve the speed of the rotation step. A user manual and API documentation are available online with the software.

Being primarily developed to automate image analysis tasks, *molyso* is a command line tool. While in most cases the standard parameters are suitable for processing, extensive graphical debug output, visualizing intermediate results, is available to the user to allow tuning of processing parameters with representative images before batch-processing large-scale data sets (cf. user manual). An example debug output is given in S5 Fig. Since segmentation performance is essential for all downstream analysis steps, an interactive preview allows for rapid checking of cell detection results of individual frames.

**Fig 4:**
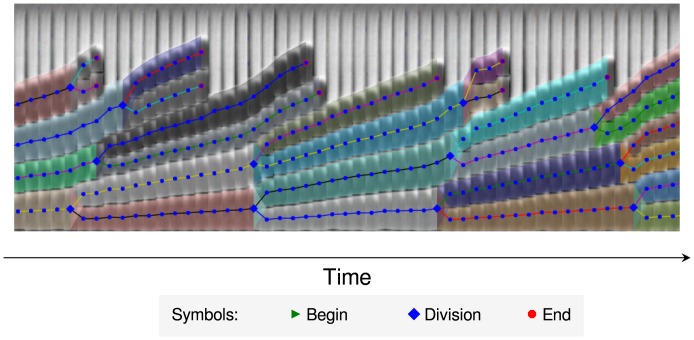
Kymograph of a time-resolved channel image collage taken from Case Study A, as produced by *molyso*. Larger kymographs and further explanation are found in the S6 Fig.

Image data is read and processed in parallel on a per-frame basis, increasing performance and reducing the memory burden (see Sec. *Computational performance evaluation* for a performance evaluation). Processing functions are separated from the (command-line) interface, allowing *molyso* to be used as a library for MM image processing as well, e.g., as part of custom processing pipelines. Batch processing of image data sets outputs tabular data containing information (i.e., cellular parameters, lineage) per cell sighting. Finally, graphical lineage information can be generated in form of a *kymograph* with overlaid lineage information (cf. Fig 4). Fluorescence data, if present, is also displayed in this output. In addition to automated batch processing, *molyso* offers a convenient *ground truth mode* for the manual analysis of division time data. In this mode, the user marks individual division events in a generated kymograph to get ground truth division times.

The automated analysis capabilities of *molyso* are evaluated with two example data sets, a cropped sequence for assessing the accuracy performance and a larger-scale data set to analyze fluorescent biosensor read-out in dependence of growth rate. In both cases, time-lapse image stacks of MM experiments with the *Corynebacterium glutamicum* are generated, an industrial platform organism for the production of various amino acids and secondary metabolites. Detailed information [28–35] on imaging setup, microfluidic device, cultivation and strains used is found in S1 Appendix. Image datasets of Case Studies A/B are available at DOI: 10.5281/zenodo.53764.

### Case study A: accuracy benchmarking the automated analysis

Arguably one of the most important phenotypic parameters in biotechnology is the growth rate (*μ*). Therefore, we first focus on deriving a benchmark measure for the doubling time *t*_*d*_ which directly translates into the growth rate (*μ* = log(2)/*t*_*d*_). For the case study, the image data set consists of a crop of only two growth channels filled with growing *C. glutamicum* cells, imaged at a 5 min interval over about 20 h. For the validation, ground truth (GT) data was generated by manual division event identification with *molyso*. Notably, GT division times have a standard deviation as well, mainly due to cellular heterogeneity and the temporal quantization by the imaging frequency.

**Fig 5:**
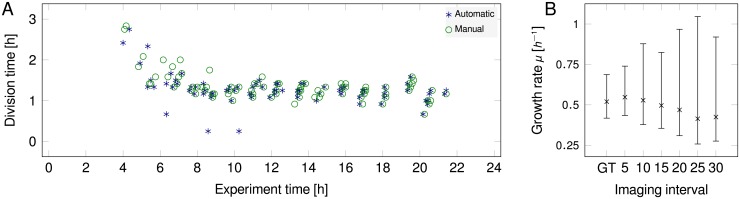
**A:** Timing of division events along with division times determined with *molyso* automatically and manually with the *ground truth mode*. Division events appear after an initial lag phase. Corresponding kymographs of the analyzed channels are shown in S6 Fig. Studying the kymographs revealed the origin of erroneously detected division events: the medium flow shifts cells quickly to the end of the channel leading to false-too-low division times. **B:** Growth rates determined for data sub-sets and GT plotted versus the imaging interval.

The automatic analysis identified 92 division events, while the GT contained one less (91). The division time determined with the automated approach is *t*_*d*,A_ = 1.27 ± 0.33 h, while the manual GT found a comparable value of *t*_*d*,GT_ = 1.33 ± 0.32 h. This results in a growth rate of *μ*_A_ = 0.55 h^−1^ that is ≈ 5.3% higher than the value for the GT (*μ*_GT_ = 0.52 h^−1^). Fig 5A shows the individual division events with division time, time of occurrence and growth rate for the automated and manual analysis, showing that the majority of automatic detections coincide with manually derived GT events. To quantify analysis precision, the Manhattan distance for paired automated and GT division events is calculated while allowing for a certain offset of division time and division age. This approach yields the following precision 79, 73, 59, 10% and recall values 80, 74, 59, 10% at max. 3, 2, 1, 0 frames offset, respectively (cf. Fig 5A).

Fig 4 shows part of a kymograph generated by *molyso*, larger tracking kymographs, as well as GT division event data, are found in S6 Fig. Example single cell traces (graphs length over time of mother cells) generated from the tabular output of *molyso* are presented in Fig 7A/7B showing an expected regular sawtooth pattern.

Often technical (e.g., number of imaged on-chip positions) or biological (exposure by illumination) practicability limit the achievable imaging frequency [36]. Because the frame-rate-to-division-time ratio is inherently connected with the tracking performance, we analyzed only every *i*^th^ frame, thus mimicking larger imaging intervals of 10, 15, 20, 25, and 30 min, respectively. For these cases the impact of the imaging frequency on the precision of the result was evaluated. Fig 5B shows resulting growth rates as compared to the GT. As expected, growth rate precision and standard deviation deteriorate with longer imaging intervals (cf. Fig 5B). Near GT results are found for imaging frequencies of 5 min with growth rates of 0.5–0.6 h^−1^. Expectedly, missing data can hardly be compensated by algorithmic (but also human) means, indicating the importance of a reasonable imaging frequency. Hence, Fig 5B provides insights to experimenters to determine the acceptable trade-off between imaging frequency and precision.

### Case Study B: microbial single cell screening

To support phenotypic single cell assays and screenings, several single-cell parameters, including their complete time-resolved ancestry are of key interest. The objective of this case study is to evaluate the automatic extraction of the available cellular parameters with temporal resolution. In turn the results are related to the growth rate at population level. As an example, a typical use case rooted in bioprocess development is selected where the spatio-temporal cell-to-cell heterogeneity is of special interest.

An engineered *C. glutamicum* l-valine production strain with a metabolite eYFP-based biosensor [30] was grown on medium containing acetate. After switching the medium to a medium lacking acetate, growth arrested while l-valine production started as indicated by an increase in fluorescent sensor signal. The imaging frequency was 15 min. In contrast, fluorescence information was captured only every hour.

**Fig 6:**
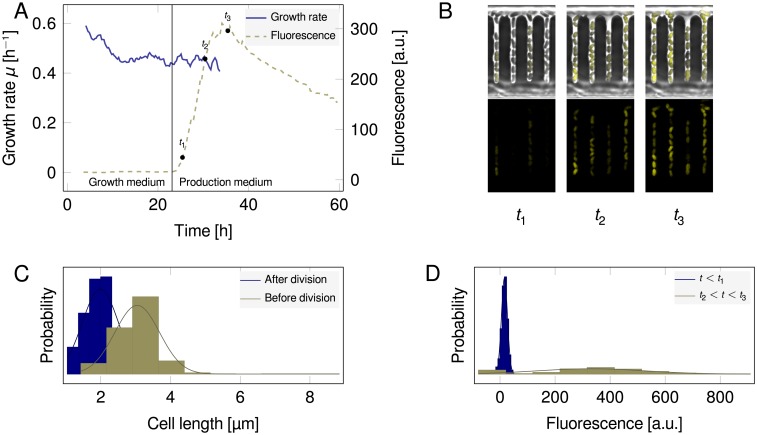
**A:** Growth rate *μ* (solid line) as well as fluorescence values (dashed line) calculated by moving average of all analyzed cells (window size 25). Cells stop growing after medium change to production medium (solid vertical line). After a slight delay the production phase starts as indicated by an increase in fluorescent biosensor read-out. A growth rate is undefined if a time point failed to produce a minimal number of division events to eliminate spurious values due to artifacts. **B**: Three time points selected from the production phase: near beginning (*t*_1_ = 25.4 h), near peak (*t*_2_ = 30.4 h) and early reduction (*t*_3_ = 35.4 h) (top row: phase contrast and fluorescence; bottom row: fluorescence only). **C**: Distribution of cell lengths before and after division during growth phase (*t* < *t*_1_) (solid lines denote fitted normal distributions). **D**: Distribution of fluorescence values during growth and production phase.

Fig 6A/6B shows the overall growth rate and snapshots of selected time points (*t*_1,2,3_). Due to growth arrest, the growth rate can no longer be determined after 30 h. To verify the automatically determined growth rates, a sample of the image data was manually analyzed with the GT mode where only events before 24 h were included.

**Fig 7:**
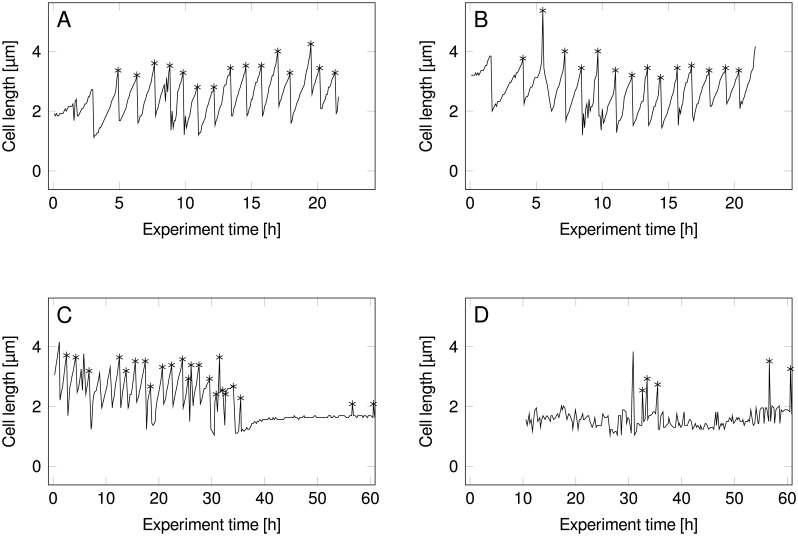
Cell length over time graphs. Asterisks denote detected cell division events. **A** and **B** are derived from Case Study A, and show high quality tracks. Good tracks show the typical sawtooth curve of a cell repeatedly growing in length, then dividing. **C** and **D** are derived from Case Study B, and show a good track, as well as a bad track. The bad track is an example for a typical artifact, e.g. produced by continuously detected top or bottom channel structure fragments. As artifact tracks differ in structure from good tracks, filtering them is straigthforward.

Taking into account only division events within rather loose physiological bounds (0.01 h^−1^ < *μ* < 1.0 h^−1^), the automated analysis yielded an average division time of *t*_*d*,A_ = 1.72 ± 0.97 h (*n* = 594), while the GT gave *t*_*d*,GT_ = 1.73 ± 0.25 h (*n* = 526), thus, both yielding a growth rate of *μ*_GT_ = *μ*_A_ = 0.40 h^−1^. For the automated analysis, however, almost fourfold higher standard deviation was found: slower image acquisition rates tend to deteriorate the tracking quality, leading to artifacts that often cancel out in the first moment but are reflected in the variance, cf. Fig 5B. Two repesentative single cell traces are shown in Fig 7C/7D, one being a good track, and one being an artifact erroneous track.

The distribution of cell lengths before and after division during growth phase (*t* ≤ *t*_1_) is shown in Fig 6C: measured length were 3.04 ± 0.64 *μ*m right before dividing and 1.99 ± 0.52 *μ*m after division. The average cell length (2.35 ± 0.66 *μ*m) is in accordance to previously published results for cells grown in minimal medium (2.40 *μ*m, [33]). In peak production phase (*t*_2_ ≤ *t* ≤ *t*_3_), cell lengths are minimally lower (2.12 ± 0.85*μ*m). Expectedly, fluorescence signals significantly increased in the production phase (330.67 ± 197.96 a.u.) as compared to the growth phase (16.07 ± 10.98 a.u., cf. Fig 6D). Fluorescence values are calculated based on the mean value of the rectangular cell bounding box. Background value is the mean of the MM structure fluorescence *between* the growth channels, and subtracted from the cell value. Notably, the fluorescence signal has a high standard deviation, pointing to a heterogeneity in l-valine production.

From the kymographs generated, samples are randomly picked and visually inspected. Despite the fact that the imaging interval was 15 min, only a small number of cells were mis-tracked. Notably, a failure in the tracking results in the registration of a division event on a later frame and, eventually, sorts itself out over time. In other words, tracking errors, if existent, are usually located near the leaves of the lineage tree.

### Computational performance evaluation

Performance evaluation was done on a workstation with 8 core Xeon E5-2650 v2 2.6 GHz CPU (with hyper threading), 64 GB RAM and SSD, under Python 3.4.3 on Windows 7, with the whole dataset from which Case Study B is derived (14,616 images from 58 positions and 252 time points, ≈90 GB). The benchmark was run for 10 times. Processing the individual images was performed in 16 parallel processes, which yielded a processing speed of 33.99 ± 0.30 frames per second. The images were processed in 430.01 ± 3.75 s. Serial cell tracking took 1,124.36 ± 50.31 s. Outputting of tabular data, information about 1,024,924 individual cell detections with 39,366 division events, took 144.97 ± 1.29 s. Optional automated visualization of cell lineages with matplotlib took 3,988.18 ± 23.50 s. Therefore, analyzing the whole dataset of the two-day experiment took approximately 28.32 ± 0.83 min to generate numerical results, or approximately 94.79 ± 0.96 min with included visualization. To see how much speed-up an automated solution can deliver compared to a semi-automatic one, the dataset of Case Study A was analyzed in molyso and with mmJ [9] in S2 Table.

## Conclusions

Data generated with the MM enable unique insights into the development of single rod-shaped bacterial cells over many generations in union with their exact ancestry. A high level of automation is essential to use this setup for large scale single-cell assays that aim to provide statistically sound measures of cellular features (e.g., morphology, fluorescence) and their development over time. We present a fully automated analysis solution, provided by the open source software *molyso*, for time-lapse image data generated by the MM. The validity of our approach and its preciseness was validated with two case studies investigating the microbe *C. glutamicum*. We demonstrated a high correspondence between manual and automated analysis for sufficient imaging frequencies. The software was tested with different mother machine design variants, such as open ended structures [12] (some examples are shown in S4 Fig) and proved itself to yield robust results. The image down-sampling study, Fig 5B in particular, can help experimenters with choosing an acceptable trade-off between imaging frequency, (photo)toxicity and precision of physiological descriptors of interest.

The vast amounts of data generated by *molyso* can be reduced to averages (e.g., growth rate or fluorescence over time) or used at single-cell resolution. The latter can be assessed, e.g., by visual investigation using the built-in kymograph visualization feature, custom post-processing (e.g. cell length versus time graphs like Fig 7), or, straightforward by conversion to *MetaXML/phyloXML* format for analysis using the lineage tree analyzer software Vizardous [37] (cf. S7 Fig). So far, *molyso* has been used to analyze over 80 days of microscopy experimentation—over 4 TB of image data—and has proven to quickly extract cellular measures in a fully automated manner. With that, our open source software contributes a platform for future developments (e.g., on-line live analysis or the extraction of individual cell images for further intracellular analyses), useful to other scientists to speed up the extraction of information from MM image data.

## Supporting Information

S1 Fig Overview of segmentation results with 2D segmentation tools. (PDF)

S2 Fig Channel detection. (PDF)

S3 Fig Cell tracking. (PDF)

S4 Fig Various mother machine structure tests. (PDF)

S5 Fig Example debug output. (PDF)

S6 Fig Ground truth / lineage reconstruction of Case Study A. (PDF)

S7 Fig Example visualization using Vizardous. (PDF)

S1 Table Cell tracking formulae table. (PDF)

S2 Table Comparison of analysis duration between molyso and mmJ. (PDF)

S1 Appendix Experimental setup, cultivation and chip design. (PDF)
