# Supplementary material for: Image-Based Single Cell Profiling: High-Throughput Processing of Mother Machine Experiments
Source: PLoS One. 2016 Sep 23;11(9):e0163453. doi: 10.1371/journal.pone.0163453 (PMC5035088; doi:10.1371/journal.pone.0163453)
Supplement: S1 Fig — (PDF) [file pone.0163453.s001.pdf]

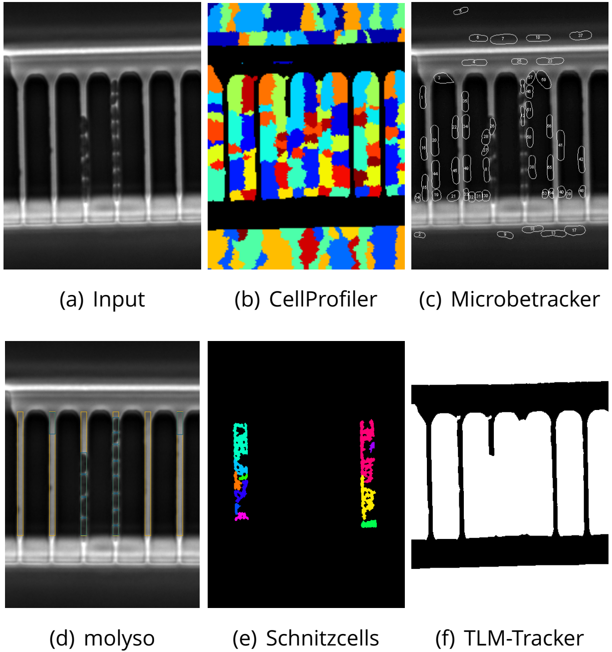

Supplementary Figure 1 (a): input image sample used for test segmentations. All tools are executed with standard parameters, results are shown in (b)-(f). (b): CellProfiler with the *IdentifyPrimaryObjects* function in mode *Auto*. The input was inverted before analysis. (c): Microbetracker; multiple configuration were found to yield similarly results. (d): molyso automatically rotates the input image. (e): Schnitzcells with the *e.coli* preset. (f): TLM-Tracker with the Chan-Vese active contours method as one example of multiple, similar performing segmentation techniques.
