# Supplementary material for: Image-Based Single Cell Profiling: High-Throughput Processing of Mother Machine Experiments
Source: PLoS One. 2016 Sep 23;11(9):e0163453. doi: 10.1371/journal.pone.0163453 (PMC5035088; doi:10.1371/journal.pone.0163453)
Supplement: S2 Fig — (PDF) [file pone.0163453.s002.pdf]

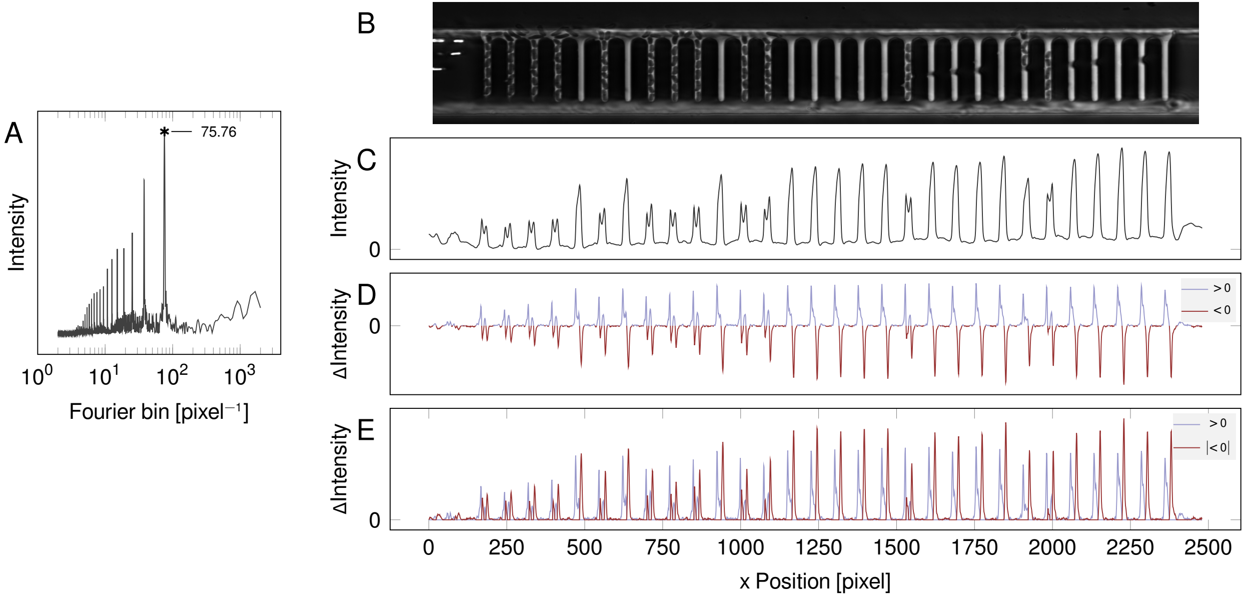

Supplementary Figure 2 **A**: An example power (absolute) spectrum of one of signals in **E**: the channel interval becomes visible as maximum (denoted with *). **B** is the rotated input image, used to calculate profile **C**, of which the discrete difference (*i.e.* the derivative) **D** is calculated. **C** can be split into positive and negative parts, which correspond to the left/right sides of the channels. **E** is the intermediate result used for further processing: the positive and (absolute) negative part (light blue/dark red) of the profile.
