# Supplementary material for: Image-Based Single Cell Profiling: High-Throughput Processing of Mother Machine Experiments
Source: PLoS One. 2016 Sep 23;11(9):e0163453. doi: 10.1371/journal.pone.0163453 (PMC5035088; doi:10.1371/journal.pone.0163453)
Supplement: S3 Fig — (PDF) [file pone.0163453.s003.pdf]

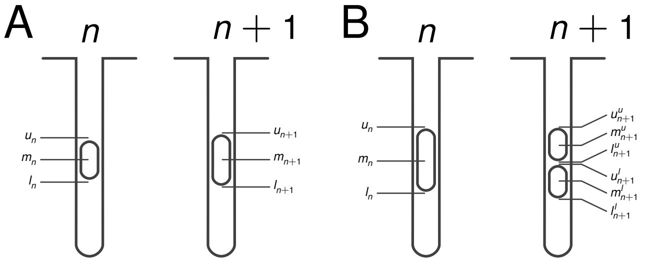

Supplementary Figure 3 Visualization of two different situations of cellular fate between two frames: Growth (**A**) and division (**B**). *n* is the time point, *u, m, l* denote the top, center and bottom positions, respectively.
