# Supplementary material for: Image-Based Single Cell Profiling: High-Throughput Processing of Mother Machine Experiments
Source: PLoS One. 2016 Sep 23;11(9):e0163453. doi: 10.1371/journal.pone.0163453 (PMC5035088; doi:10.1371/journal.pone.0163453)
Supplement: S4 Fig — (PDF) [file pone.0163453.s004.pdf]

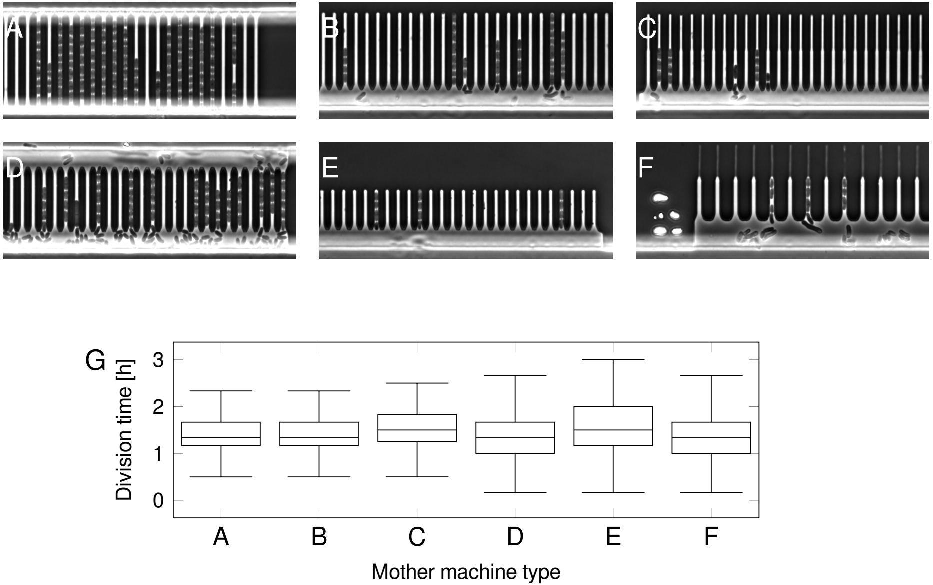

Supplementary Figure 4 Several mother machine designs were tested with *molyso*. Designs (**A**-**F**) were on the same PDMS device and cells were cultured under identical conditions. **G** shows box plots of the division time distribution. Automated analysis with the software was successful in all cases. Note that **A** and **D** are open-ended designs, which allow for continuous cultivation without an aging mother cell (chemostat conditions). Only **C** with **A** or **F**, respectively, showed a statistical significant ($p < 0.05$) difference. Besides that, the software delivered comparable division times for all designs, as expected under identical cultivation conditions. The sample sizes (division event counts) were: **A**: $n = 672$, **B**: $n = 249$, **C**: $n = 86$, **D**: $n = 425$, **E**: $n = 45$, **F**: $n = 58$.
