# Supplementary material for: Image-Based Single Cell Profiling: High-Throughput Processing of Mother Machine Experiments
Source: PLoS One. 2016 Sep 23;11(9):e0163453. doi: 10.1371/journal.pone.0163453 (PMC5035088; doi:10.1371/journal.pone.0163453)
Supplement: S5 Fig — (PDF) [file pone.0163453.s005.pdf]

5 Example debug output

The following 4 pages contain debug output as created by the `-debug` option, taken verbatim. Visualizing individual processing steps can help to find optimal parameters for certain tunables, and helps troubleshooting. Individual outputs comprise: input image, verbatim; input image, rotated; a plot of the horizontal profile; a plot of the vertical profile; the differentials of the horizontal profile used for channel detection; a spectrum (logarithmic plot of the Fourier transform magnitude) of the positive parts of the differential; a spectrum of the negative parts; the detected channels on the original image; the detected channels on the rotated image; for each of the 30 channels, plots of the horizontal smoothed baseline corrected profile of the channel image, as well as a thresholded profile used for empty channel detection, the channel image and its Otsu binarization, as well as cell candidates; a final image showing the rotated image with channel and cell information overlayed.

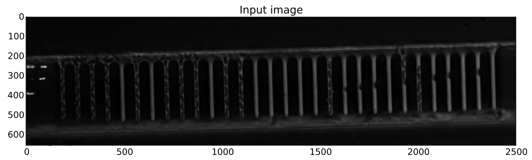
Input image

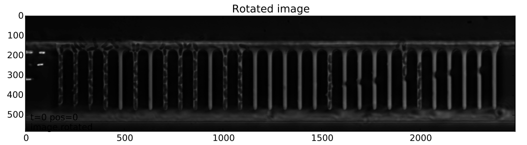
Rotated image
t=0 pos=0

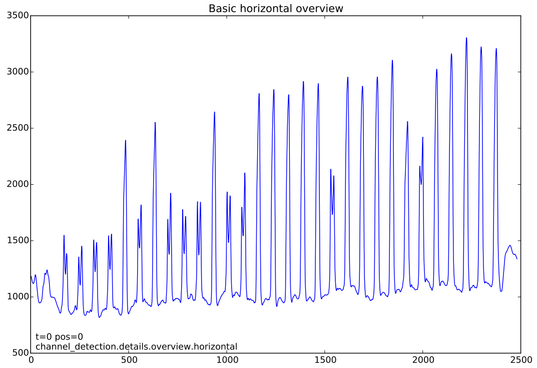
Basic horizontal overview
t=0 pos=0
channel_detection.details.overview.horizontal

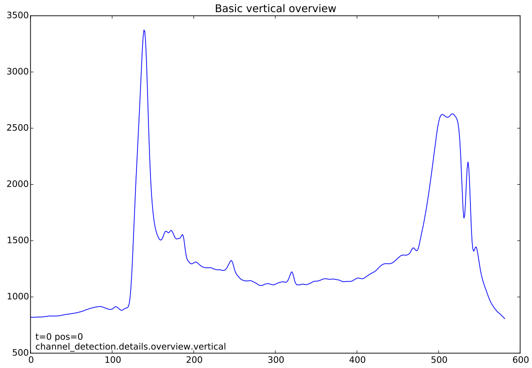
Basic vertical overview
t=0 pos=0
channel_detection.details.overview.vertical

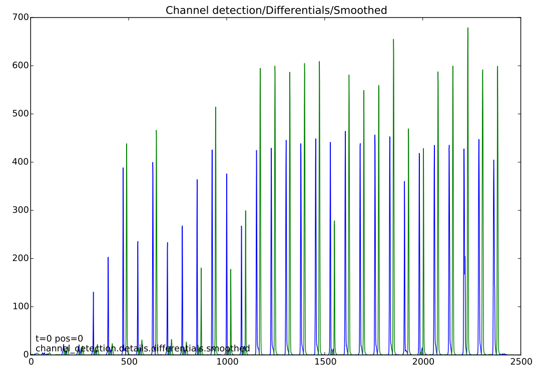
Channel detection/Differentials/Smoothed
t=0 pos=0

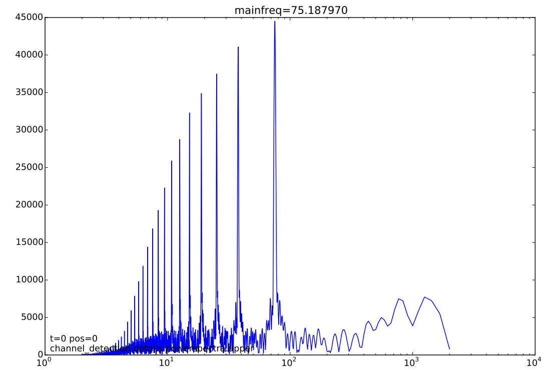
mainfreq=75.187970
t=0 pos=0

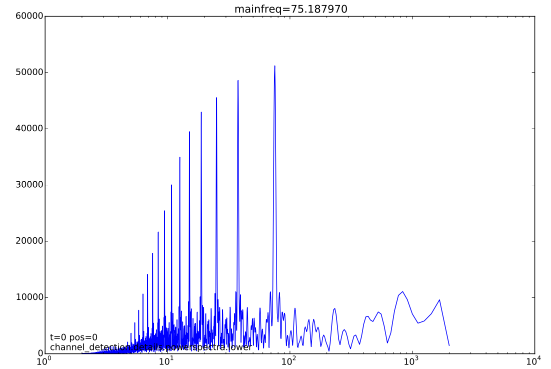
mainfreq=75.187970
t=0 pos=0

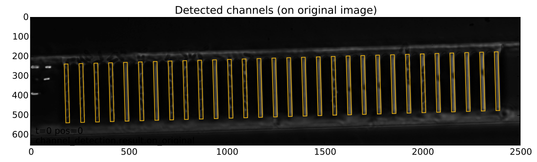
Detected channels (on original image)
t=0 pos=0

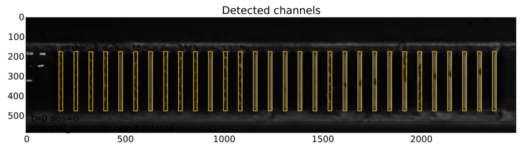
Detected channels
t=0 pos=0

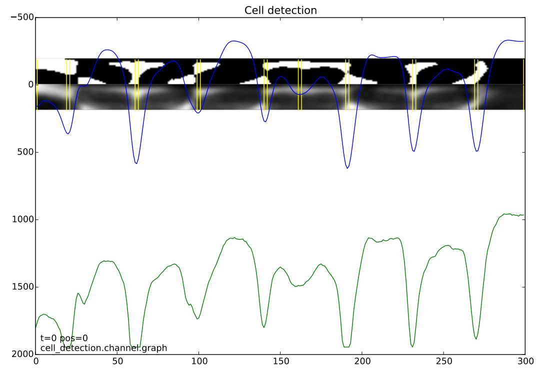
Cell detection
t=0 pos=0
cell_detection.channel.graph

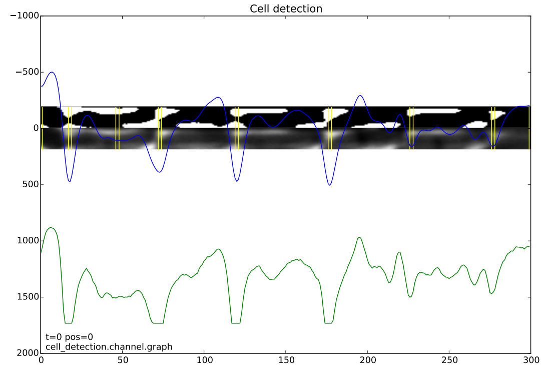
Cell detection
−1000 −500 0 500 1000 1500 2000
0 50 100 150 200 250 300
t=0 pos=0
cell_detection.channel.graph

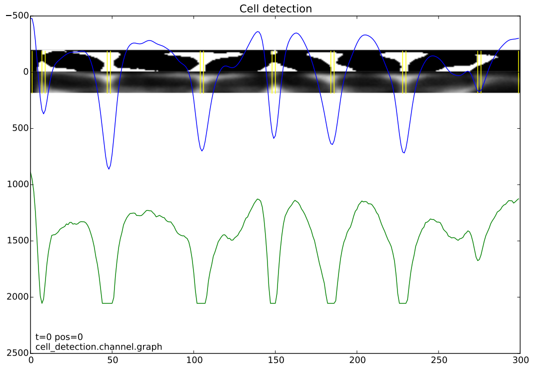
Cell detection
−500 0 500 1000 1500 2000 2500
0 50 100 150 200 250 300
t=0 pos=0
cell_detection.channel.graph

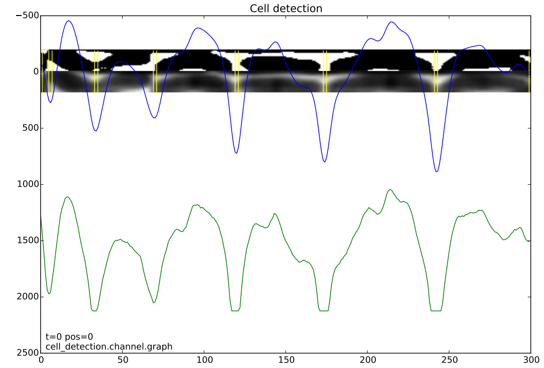
Cell detection
−500 0 500 1000 1500 2000 2500
0 50 100 150 200 250 300
t=0 pos=0
cell_detection.channel.graph

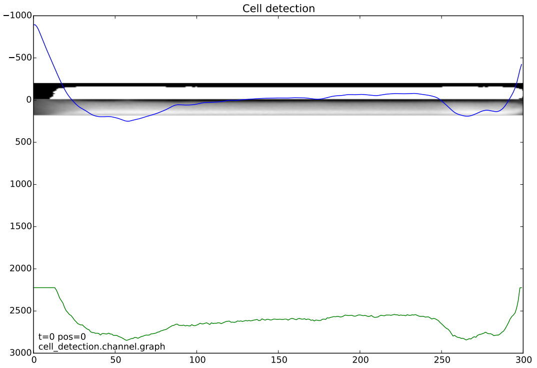
Cell detection
−1000 −500 0 500 1000 1500 2000 2500 3000
0 50 100 150 200 250 300
t=0 pos=0
cell_detection.channel.graph

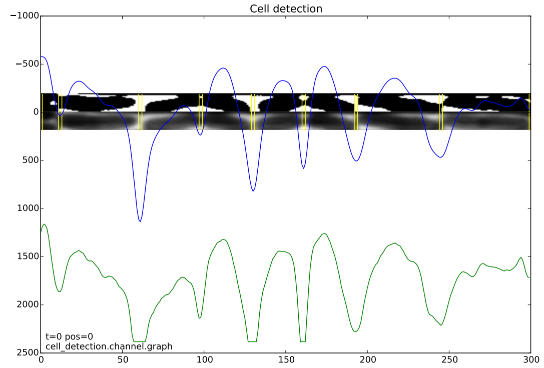
Cell detection
−1000 −500 0 500 1000 1500 2000 2500
0 50 100 150 200 250 300
t=0 pos=0
cell_detection.channel.graph

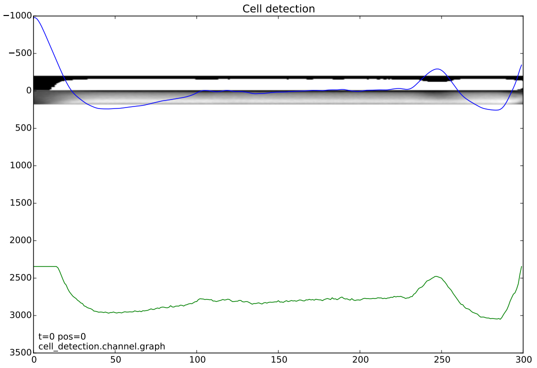
Cell detection
−1000 −500 0 500 1000 1500 2000 2500 3000 3500
0 50 100 150 200 250 300
t=0 pos=0
cell_detection.channel.graph

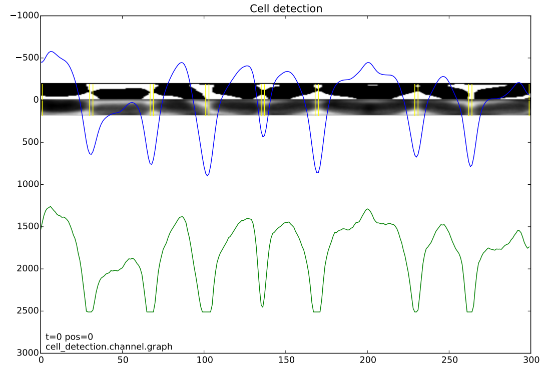
Cell detection
−1000 −500 0 500 1000 1500 2000 2500 3000
0 50 100 150 200 250 300
t=0 pos=0
cell_detection.channel.graph

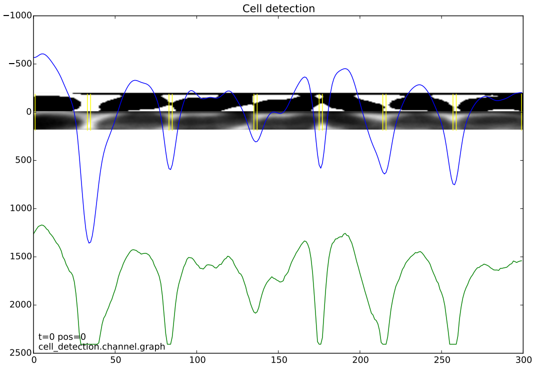
Cell detection
−1000 −500 0 500 1000 1500 2000 2500
0 50 100 150 200 250 300
t=0 pos=0
cell_detection.channel.graph

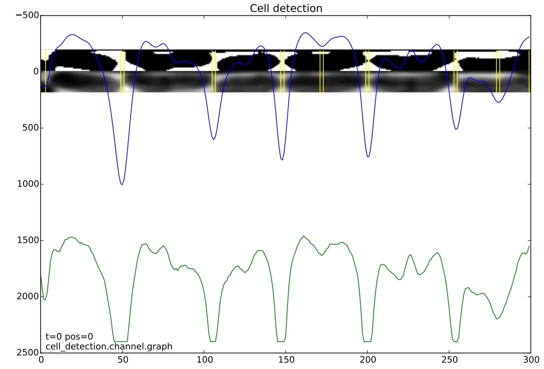
Cell detection
−500 0 500 1000 1500 2000 2500
0 50 100 150 200 250 300
t=0 pos=0
cell_detection.channel.graph

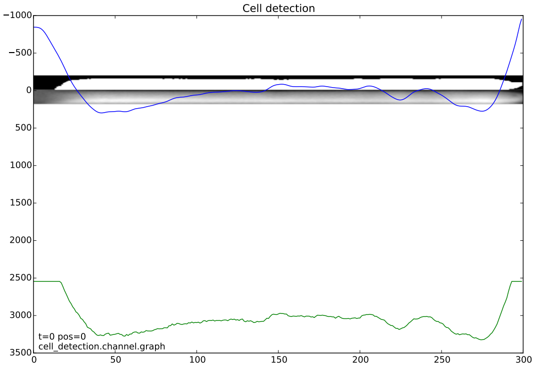
Cell detection
−1000 −500 0 500 1000 1500 2000 2500 3000 3500
0 50 100 150 200 250 300
t=0 pos=0
cell_detection.channel.graph

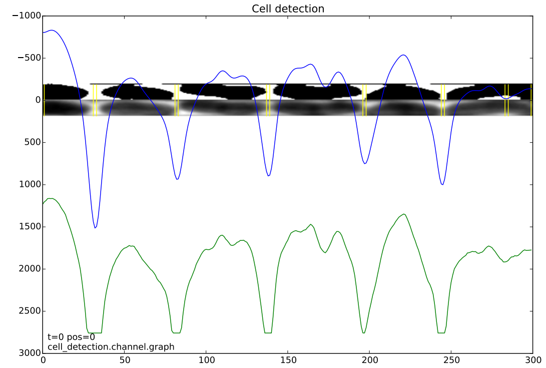
Cell detection
t=0 pos=0
cell_detection.channel.graph

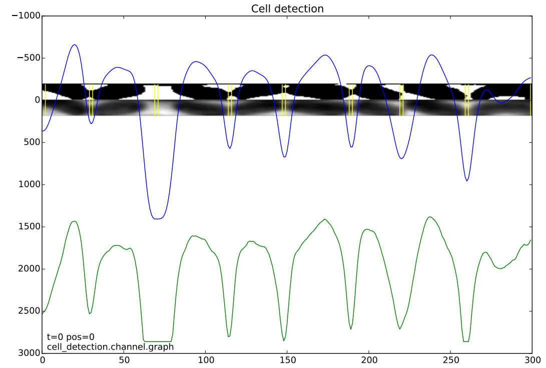
Cell detection
t=0 pos=0
cell_detection.channel.graph

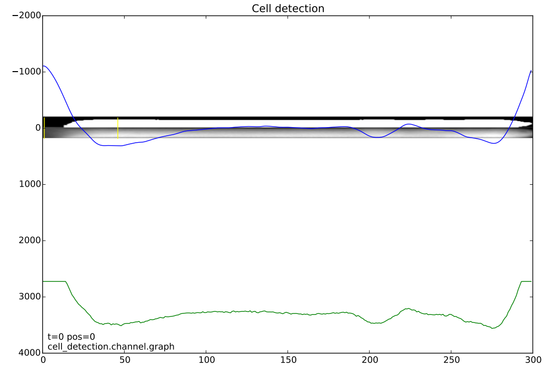
Cell detection
t=0 pos=0
cell_detection.channel.graph

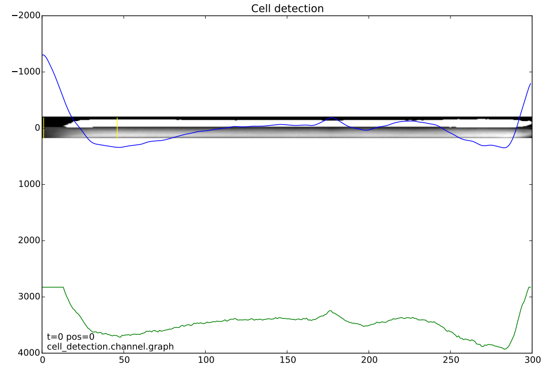
Cell detection
t=0 pos=0
cell_detection.channel.graph

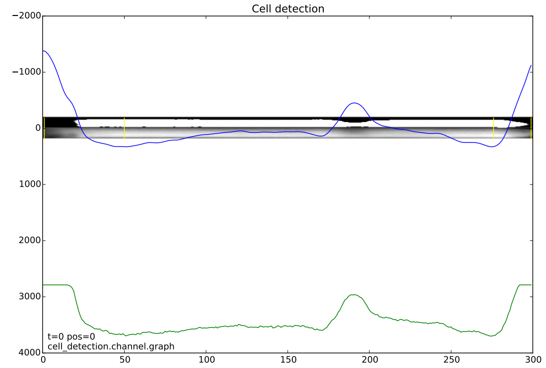
Cell detection
t=0 pos=0
cell_detection.channel.graph

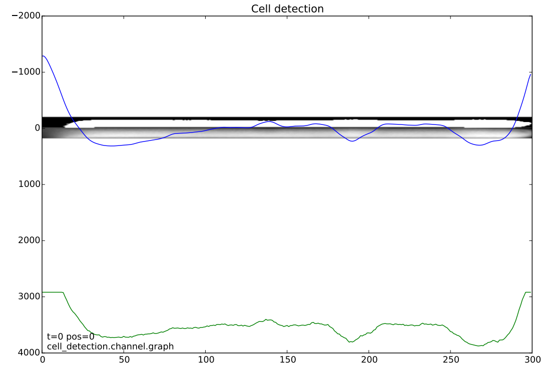
Cell detection
t=0 pos=0
cell_detection.channel.graph

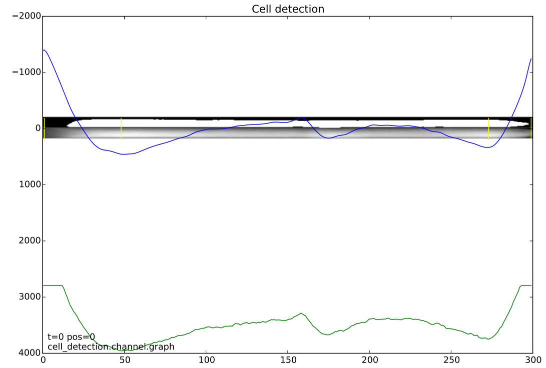
Cell detection
t=0 pos=0
cell_detection.channel.graph

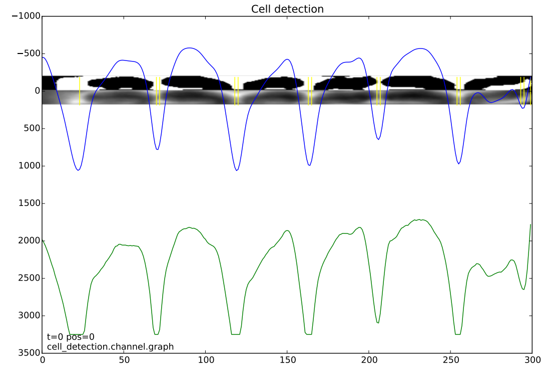
Cell detection
t=0 pos=0
cell_detection.channel.graph

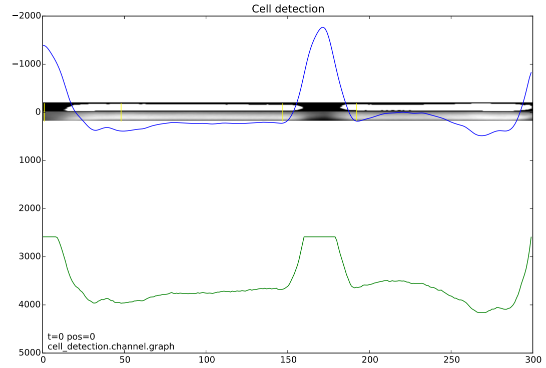
Cell detection
t=0 pos=0
cell_detection.channel.graph

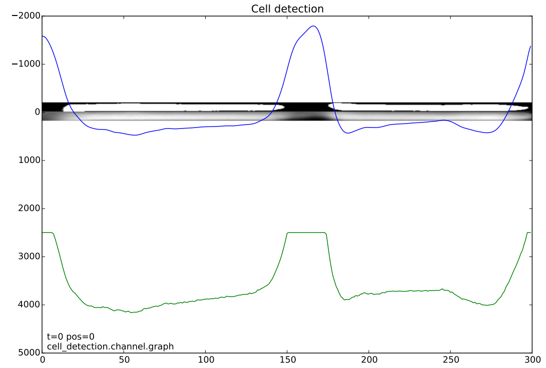
Cell detection
t=0 pos=0
cell_detection.channel.graph

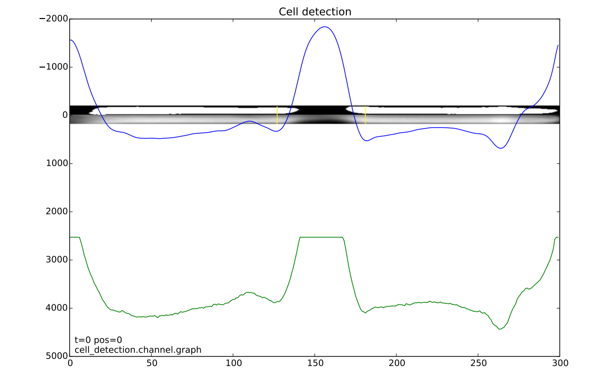
Cell detection
t=0 pos=0
cell_detection.channel.graph

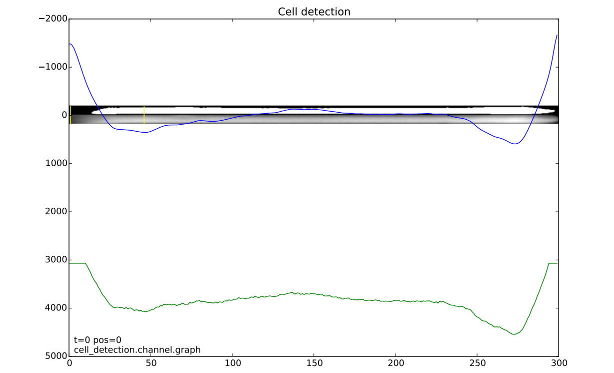
Cell detection
t=0 pos=0
cell_detection.channel.graph

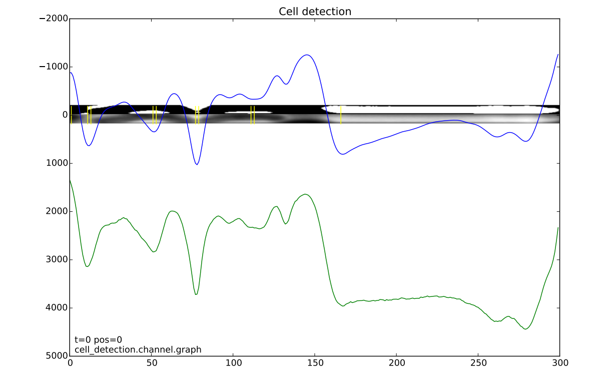
Cell detection
t=0 pos=0
cell_detection.channel.graph

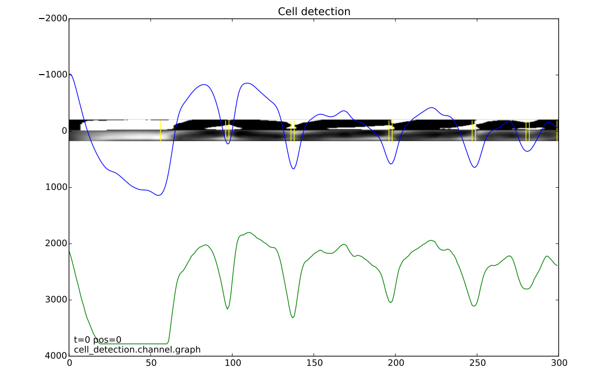
Cell detection
t=0 pos=0
cell_detection.channel.graph

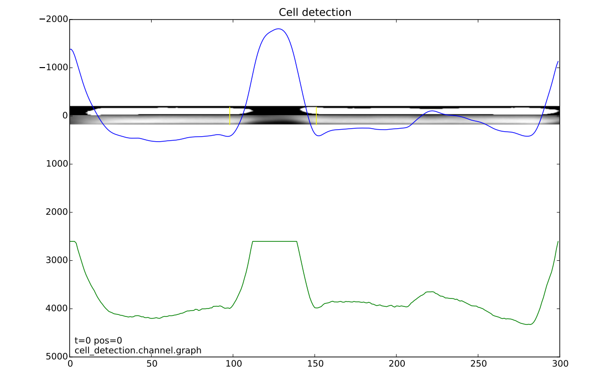
Cell detection
t=0 pos=0
cell_detection.channel.graph

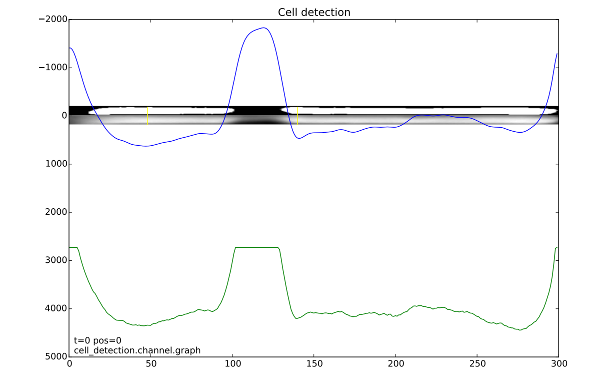
Cell detection
t=0 pos=0
cell_detection.channel.graph

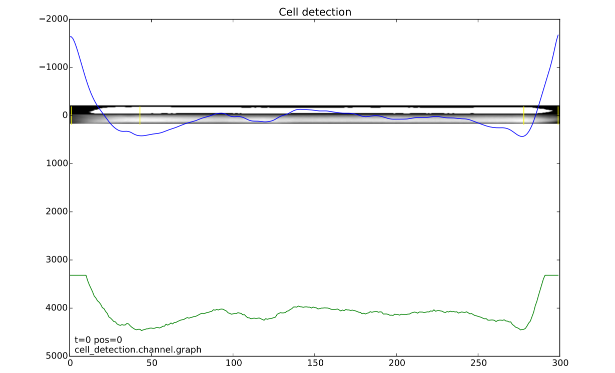
Cell detection
t=0 pos=0
cell_detection.channel.graph

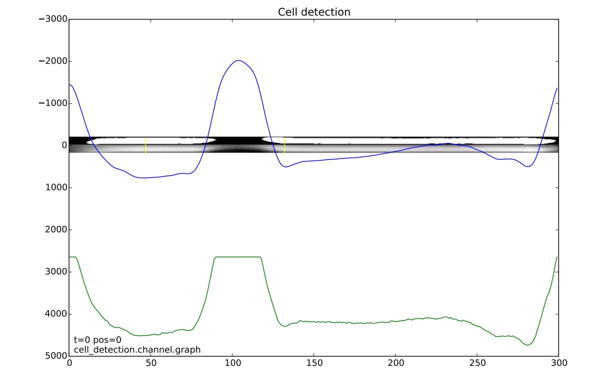
Cell detection
t=0 pos=0
cell_detection.channel.graph

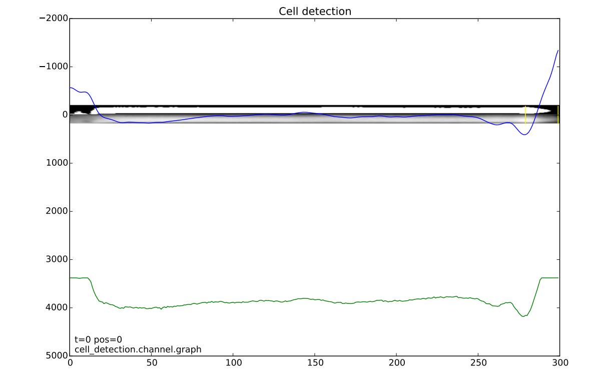
Cell detection
t=0 pos=0
cell_detection.channel.graph

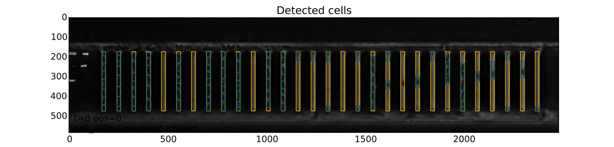
Detected cells
t=0 pos=0
