# Supplementary material for: Image-Based Single Cell Profiling: High-Throughput Processing of Mother Machine Experiments
Source: PLoS One. 2016 Sep 23;11(9):e0163453. doi: 10.1371/journal.pone.0163453 (PMC5035088; doi:10.1371/journal.pone.0163453)
Supplement: S6 Fig — (PDF) [file pone.0163453.s006.pdf]

6 Ground truth / lineage reconstruction of small example

The following page contains the two kymographs constructed from the small example. Of the two channels, the upper kymograph is just an assembly of images, with the division events added manually, and serves as ground truth data, and the lower image is automatically generated by the software. The scaling of the x axis is different however: in the kymograph assembly, channel images were assembled with identical pixel sizes (isopixel). In the automatic tracking, individual channel images were scaled according to the time slot they took (isochronous). Information on the kymographs is multi-layered: on the channel images each detected cell is denoted by a translucent gray block. On top, lineage information is indicated both in larger stripes, as well as lines through the centroids of the cells. Each cell track is marker-colored to aid visual analysis. Track starts/ends as well as detected division events are marked with special symbols. If fluorescence information is present, each cell's centroid is annotated by a color-coded point coding for the fluorescence information.

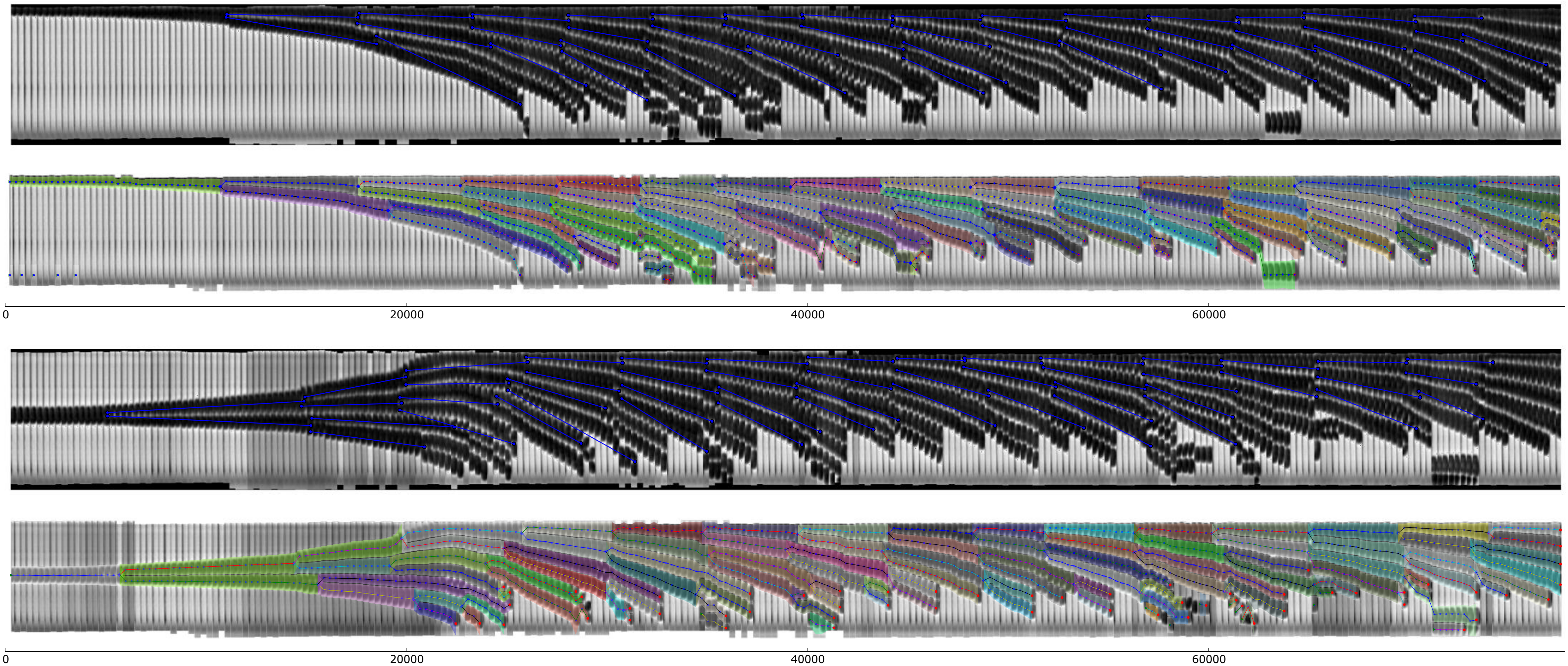
0
20000
40000
60000
0
20000
40000
60000
