# Supplementary material for: Image-Based Single Cell Profiling: High-Throughput Processing of Mother Machine Experiments
Source: PLoS One. 2016 Sep 23;11(9):e0163453. doi: 10.1371/journal.pone.0163453 (PMC5035088; doi:10.1371/journal.pone.0163453)
Supplement: S7 Fig — (PDF) [file pone.0163453.s007.pdf]

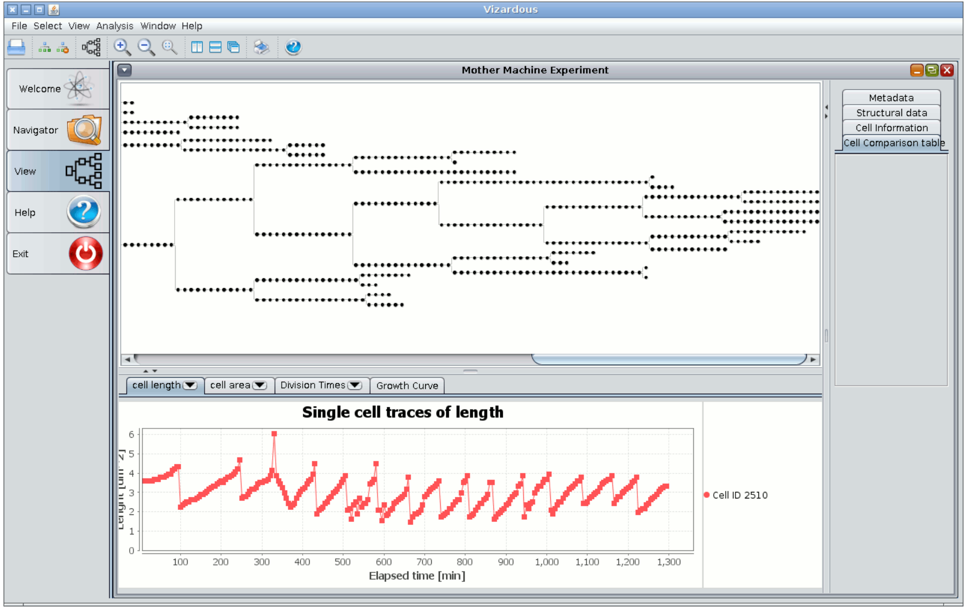

Supplementary Figure 7 Using the included tool `molyso2vizardous.py`, the tabular output of *molyso* can be converted to Vizardous-compatible *MetaXML/phyloXML* [?]. Vizardous allows for lineage tree visualization and cellular parameter analysis, shown *e.g.*, a trace of a mother cell length over time (derived from case study A).
