# Supplementary material for: Image-Based Single Cell Profiling: High-Throughput Processing of Mother Machine Experiments
Source: PLoS One. 2016 Sep 23;11(9):e0163453. doi: 10.1371/journal.pone.0163453 (PMC5035088; doi:10.1371/journal.pone.0163453)
Supplement: S1 Table — (PDF) [file pone.0163453.s008.pdf]

Supplementary Table 1 Formulae and symbols used in the cost calculations, as well as their units and descriptions. Individual cellular positional parameters used are explained in the supplementary cell tracking figure. The costs $\boldsymbol{c_N}$ for a new, and $\boldsymbol{c_L}$ for a lost cell, are high and static. The costs $\boldsymbol{c_S}$ for the same cell or $\boldsymbol{c_D}$ for a division event, are dependent on distance traveled and elongation. $\mathbf{SMA}_{\boldsymbol{w}}$ denotes the simple moving average with window width $\boldsymbol{w}$. $\boldsymbol{R}$, denotes the ramp function, which equals its argument for positive arguments, and is zero otherwise.

**Symbol**	**Unit**	**Description**	**Formula**
u	μm	Upper boundary of cell	—
m	μm	Position of cell centroid	—
l	μm	Lower boundary of cell	—
t_n	s	Absolute time of frame n	—
v	μm·s^{-1}	Cell movement speed	$\frac{\Delta m}{\Delta t}$
r	μm·s^{-1}	Cell elongation rate	$\frac{\Delta}{\Delta t}\lvert u-l\rvert$
$s_{u,l}$	μm	Expected shift of cell positions from n to $n+1$	$\left(\mathrm{SMA}_5(v) \pm \frac{1}{2}\cdot\mathrm{SMA}_5(r)\right)\cdot(t_{n+1}-t_n)$
d_{S}	μm	Difference between expected & actual length (same-cell)	$\lvert l_{n+1}-u_{n+1}\rvert - \lvert l_n-u_n\rvert - \mathrm{SMA}_5(r)\cdot(t_{n+1} -$ [illegible]
d_{D}	μm	Difference between expected & actual lengths (cell division)	$\lvert l_n-u_n\rvert + \mathrm{SMA}_5(r)\cdot(t_{n+1}-t_n) - \lvert l^u_{n+1}-u^u_{n+1}\rvert - \lvert l^l_{n+1}-u^l_{n+1}\rvert$
$p_{d_{\mathsf{S}}}$	—	Penalty summand for unexpected cell length (same-cell)	$R(-5\cdot d_{\mathsf{S}})$
$p_{d_{\mathsf{D}}}$	—	Penalty summand for unexpected cell lengths (cell division)	$R(5\cdot d_{\mathsf{D}})$
c_{N}	—	Cost for a new cell	10^6
c_{L}	—	Cost for a lost cell	10^6
c_{S}	—	Cost for a tracked (same) cell	$\frac{1}{2}\cdot(\lvert u_n+s_u-u_{n+1}\rvert + \lvert l_n+s_l-l_{n+1}\rvert) - p_{d_{\mathsf{S}}}$
c_{D}	—	Cost for a division event	$\frac{1}{2}\cdot\left(\lvert u_n+s_u-u^u_{n+1}\rvert + \lvert m_n-l^u_{n+1}\rvert + \lvert m_n-u^l_{n+1}\rvert + \lvert l_n-l^l_{n+1}\rvert\right) + p_{d_{\mathsf{D}}}$
