# Supplementary material for: Image-Based Single Cell Profiling: High-Throughput Processing of Mother Machine Experiments
Source: PLoS One. 2016 Sep 23;11(9):e0163453. doi: 10.1371/journal.pone.0163453 (PMC5035088; doi:10.1371/journal.pone.0163453)
Supplement: S2 Table — (PDF) [file pone.0163453.s009.pdf]

Supplementary Table 2 Comparison of automated and semi-automated image analysis of mother machine data with molyso and mmJ [9], respectively, using the image dataset of Case Study A.

Step	molyso	(Comment)	mmJ	(Comment)
External preparation – Registration	N/A		8m 44s	[1]
External preparation – Crop/Rotation	N/A		45s	[2]
Registration	21s			[3]
Channel detection				[4]
Cell detection			30s	
Correction		[5]	8m 52s	[6]
Tracking			20s	
Result output		[7]		[8]
Result output (graphical)	7s		N/A	
Σ	28s		19m 11s	

[1] We found many registration tools to fail at processing MM data;
we used the Fiji plugin "Descriptor based stack registration".
[2] Manual
[3] mmJ's channel detection features integrated registration. However, it did not work in our case.
[4] Manual marking necessary
[5] Manual correction is currently not possible with molyso.
[6] Misdetections on every frame, we corrected only the mother cells.
[7] molyso outputs information about all cells.
[8] mmJ only outputs information of the mother cells.
