# Supplementary material for: Image-Based Single Cell Profiling: High-Throughput Processing of Mother Machine Experiments
Source: PLoS One. 2016 Sep 23;11(9):e0163453. doi: 10.1371/journal.pone.0163453 (PMC5035088; doi:10.1371/journal.pone.0163453)
Supplement: S1 Appendix — (PDF) [file pone.0163453.s010.pdf]

1 Experimental setup, cultivation and chip design

1.1 Experimental setup & image acquisition

For imaging a Nikon Eclipse Ti inverted microscope (Nikon Corporation, Tokyo, Japan) using a Nikon Plan Apo λ 100$\times$ phase contrast objective and an Andor Neo (Andor Technology plc., Belfast, UK) camera was used. If applicable, fluorescence excitation was performed with a Nikon Intensilight and an eYFP filter (EX 520/30nm DM 510 nm, EM 540/20 nm; AHF Analysentechnik AG, Tübingen, Germany). Image stacks were acquired using the NIS-Elements (Laboratory Imaging s.r.o, Prague, Czech Republic) software in the `.nd2` format. For further details about the hardware setup for imaging we refer to Helfrich et al.[28]

The microfluidic device, a custom MM design (see Fig. for a design schematic), was mounted to the microscope, and loaded with concentrated cell suspension. To increase trapping efficiencies, the techniques described in Probst et al.[29] were applied. Nutrient media perfusion was perfomed with a precision syringe system (neMESYS, cetoni GmbH, Korbußen, Germany). The whole microscope was incubated at 30 °C with a microscope incubator (PeCon GmbH, Erbach, Germany).

The data set for case study A is taken from an experiment with a SOS reporter *Corynebacterium glutamicum* strain [28], while for case study B a production strain with L-valine productivity fluorescent biosensor is used [30]. The image stacks are available as supporting data. For image analysis default processing parameters were used (cf. user manual).

1.2 Strains used

Two different strains of *Corynebacterium glutamicum* ATCC13032 were used, for case study A, the strain ATCC 13032::P_{recA}-venus pJC1-P_{lys}-crimson was used, as described in Helfrich et al. [28]. The strain contains fluorescent biosensors for cellular SOS response (venus) and phage induction (crimson). The fluorescent information is however not used in the case study, where it serves *in lieu* of a wild type. For case study B, the strain ATCC 13032 Δ*aceE* Δ*pqo* Δ*pgi* [31] Lrp-Sensor [30] was cultivated, a L-valine production strain with a fluorescent biosensor for assessing single cell L-valine production. The strain growts in medium supplemented with acetate, and starts production in acetate-deficient medium, while stopping to grow.

1.3 Bacterial cultivation

For precultivation, the particular strain was inoculated at 30 °C either directly in CGXII [32, 33] medium from cryoconserved stocks (Roti-Store cryo vials, Carl Roth GmbH, Karlsruhe, Germany), or via a pre-cultivation in BHI medium from glycerol stocks. Once the culture had reached an OD_{600} 0.5–1, it was concentrated approximately 30–40$\times$ by centrifugation (≈14000 g, 1 min, supernatants discarded), and used for microfluidic chip seeding.

1.4 Chip structure

The microfluidic device (cf. Fig) was fabricated according to Grünberger et al. [34]. The structure contains modifications according to Kohlheyer et al. [35].

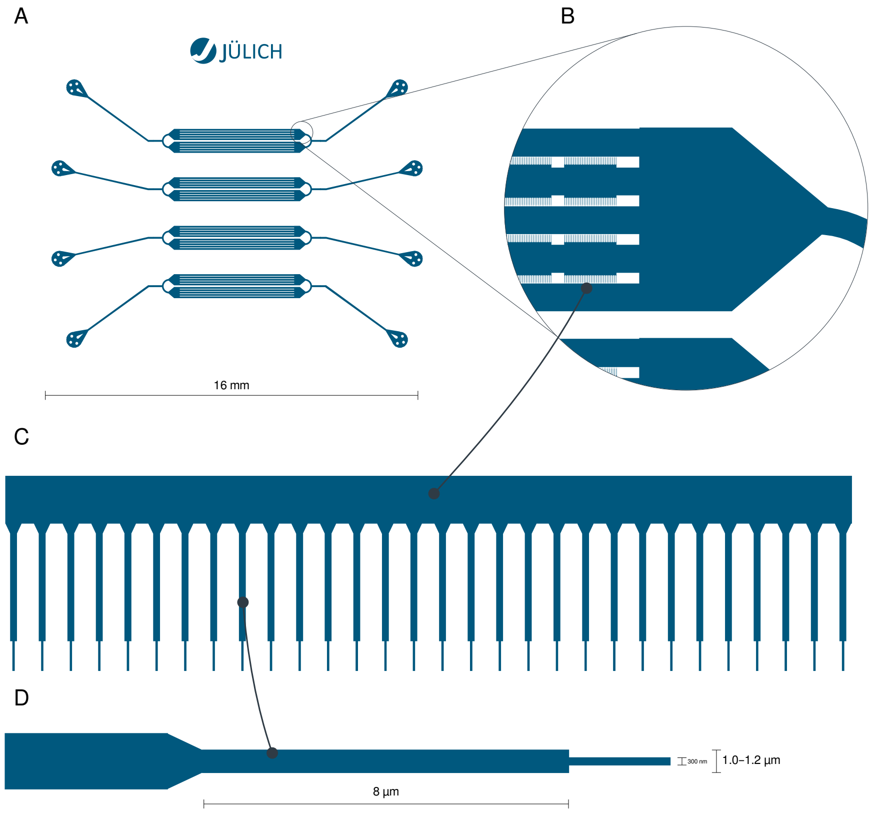

Appendix Figure Schematic overview of the chip structure. The overall chip design (**A**) consists of four parallel channels which can be used independently. Closeup **B** shows that each channel consists of a pair of smaller channels which in turn contain four rows of 26 mother machine gutter structures (**C**). Each mother machine structure contains 30 growth channels (**D**). Small sub-micrometer connections to adjacent channels allow for minimal flow through the growth channels, greatly enhancing seeding efficiency.
